# Supplementary material for: Preparation of Thermoplastic Cellulose Esters in [mTBNH][OAC] Ionic Liquid by Transesterification Reaction
Source: Polymers (Basel). 2023 Oct 3;15(19):3979. doi: 10.3390/polym15193979 (PMC10575218; doi:10.3390/polym15193979)
Supplement: Supplementary file 1 [file polymers-15-03979-s001.zip › polymers-2622951-supplementary.pdf]

*Supporting information*

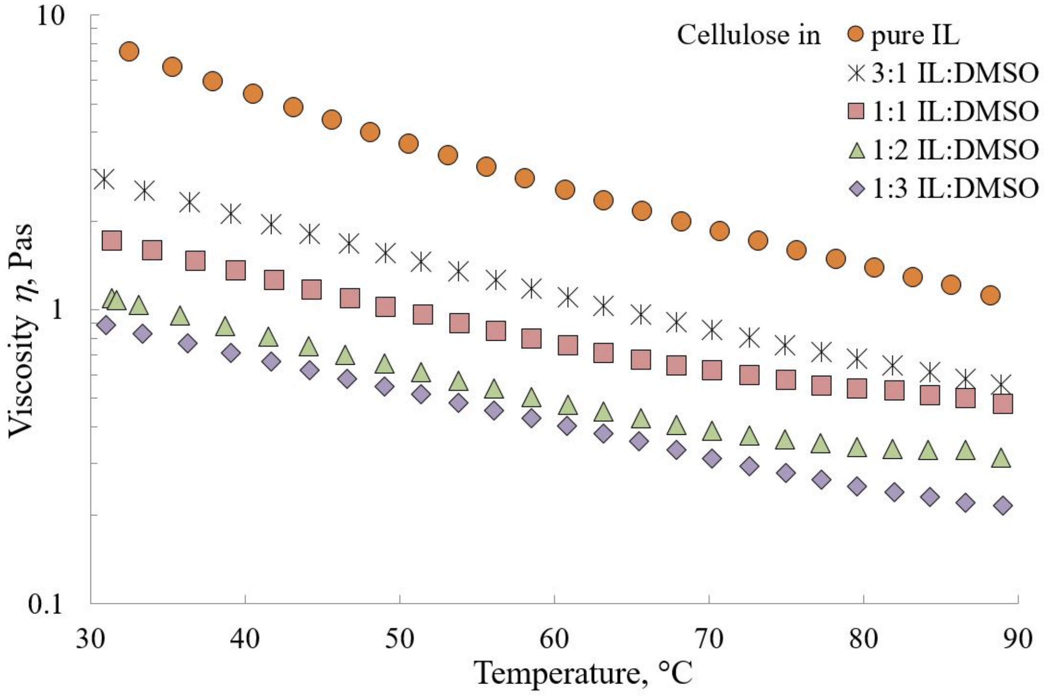


**Figure S1.** Temperature dependence of viscosity of cellulose solution in IL:DMSO binary solvent at various IL:DMSO ratios. The concentration of all solutions is 2 wt%.

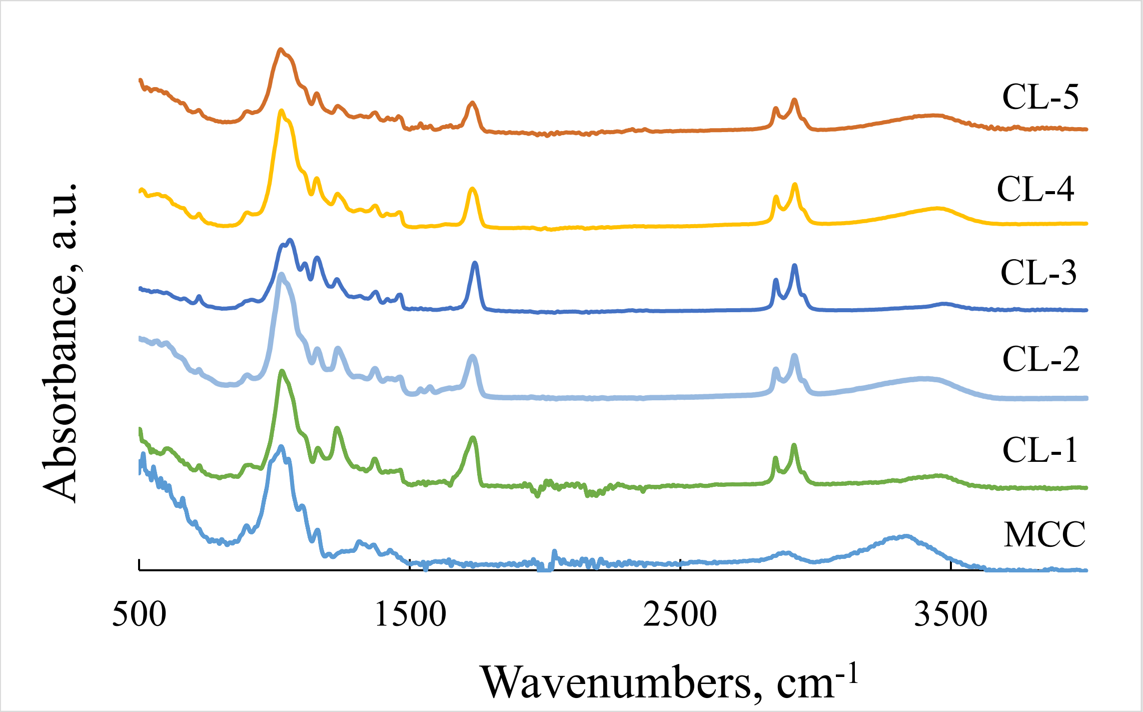


**Figure S2.** FTIR spectra of MCC and cellulose laurates obtained in IL:DMSO mixture of various ratios.

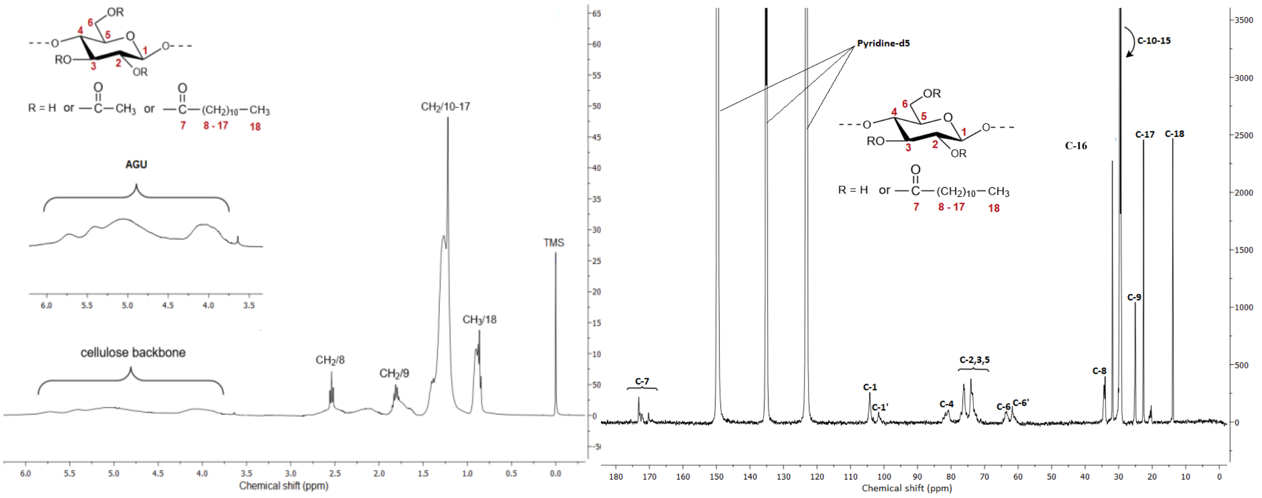


**(a)** **(b)**

**Figure S3.** (a) $^{1}H$ NMR spectrum of Cellulose Laurate CL-3 in Pyridine-$d_5$ (500 MHz, 25°C); (b) Full $^{13}C$ NMR spectrum of Cellulose Laurate CL-3 in Pyridine-$d_5$ (125 MHz, 80 °C).

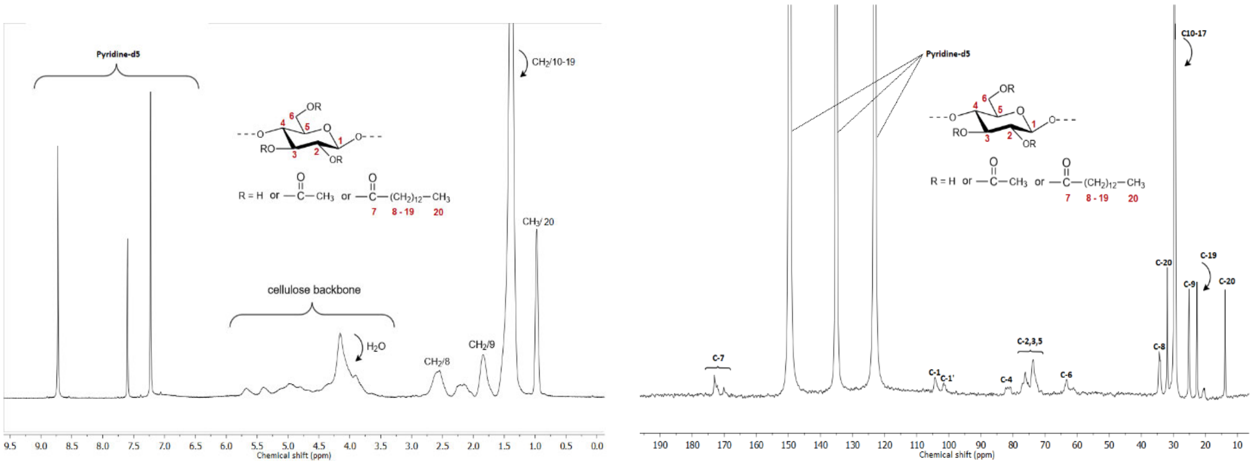


**(a)** **(b)**

**Figure S4.** (a) $^{1}H$ NMR spectrum of Cellulose Myristate in Pyridine-$d_5$ (500 MHz, 80°C); (b) $^{13}C$ NMR spectrum of Cellulose Myristate in Pyridine-$d_5$ (125 MHz, 80 °C).

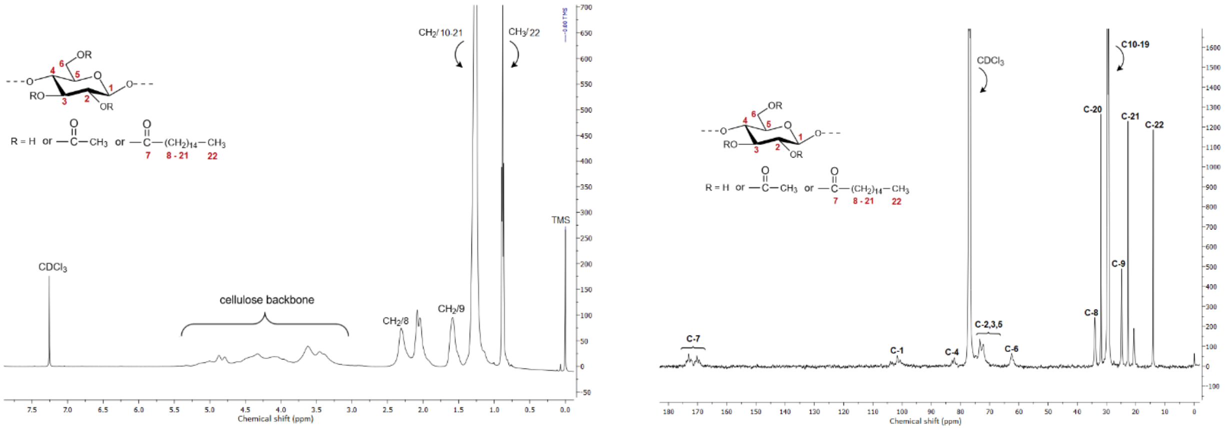


**(a)** **(b)**

**Figure S5.** (a) $^{1}H$ NMR spectrum of Cellulose Palmitate CP-3 in Chloroform-d (500 MHz, 25°C); (b) $^{13}C$ NMR spectrum of Cellulose Palmitate CP-3 in Chloroform-d (125 MHz, 40 °C).

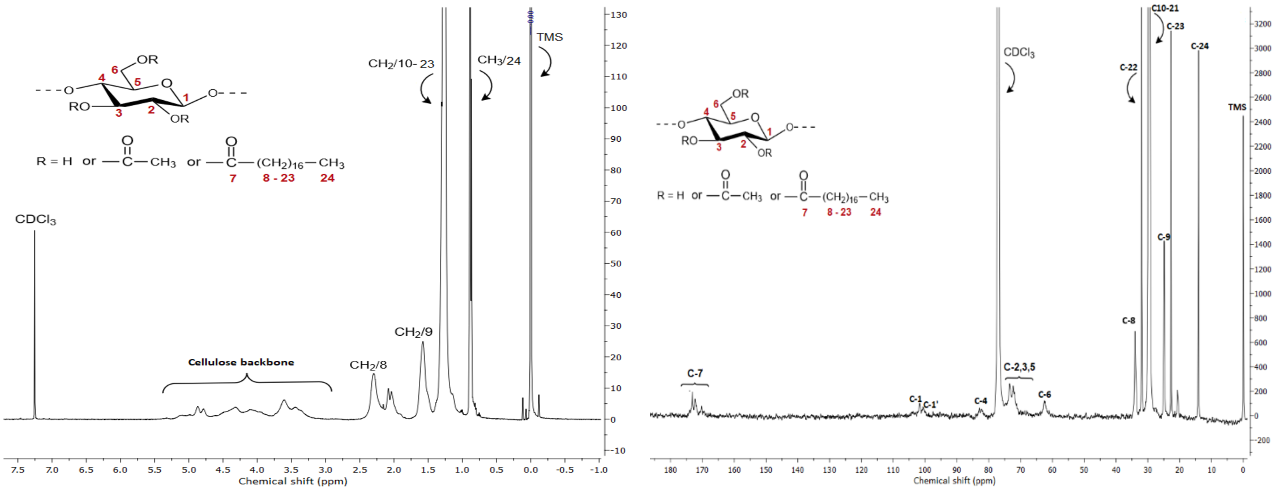


**(a)** **(b)**

**Figure S6.** (a) $^{1}H$ NMR spectrum of Cellulose Stearate CS-2 in Chloroform-d (500MHz, 40°C); (b) $^{13}C$ NMR spectrum of Cellulose Stearate CS-2 in Chloroform-d (125 MHz, 40 °C).

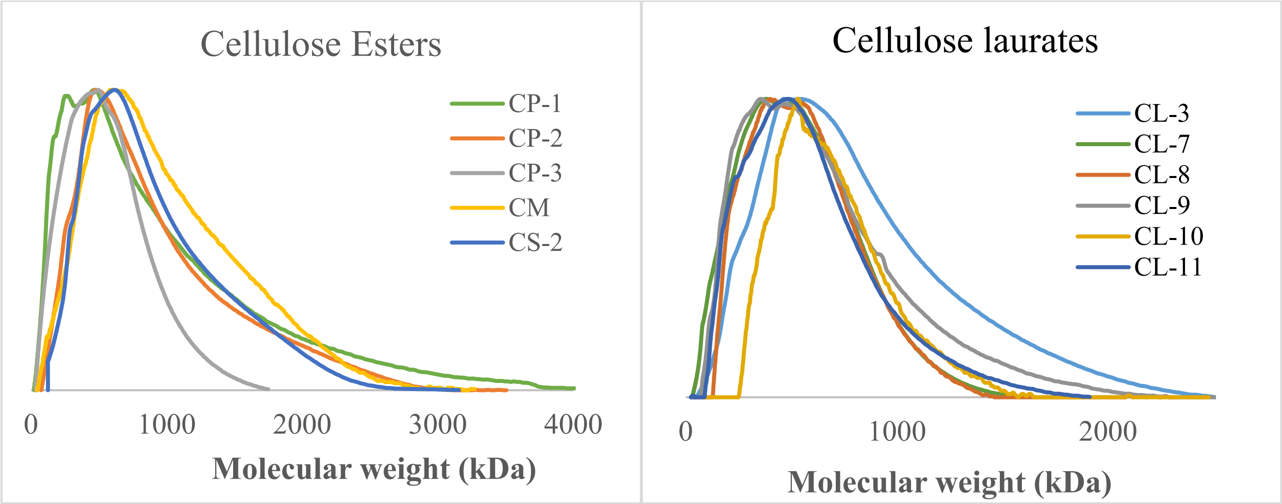


**Figure S7.** Molar mass distribution of cellulose esters.

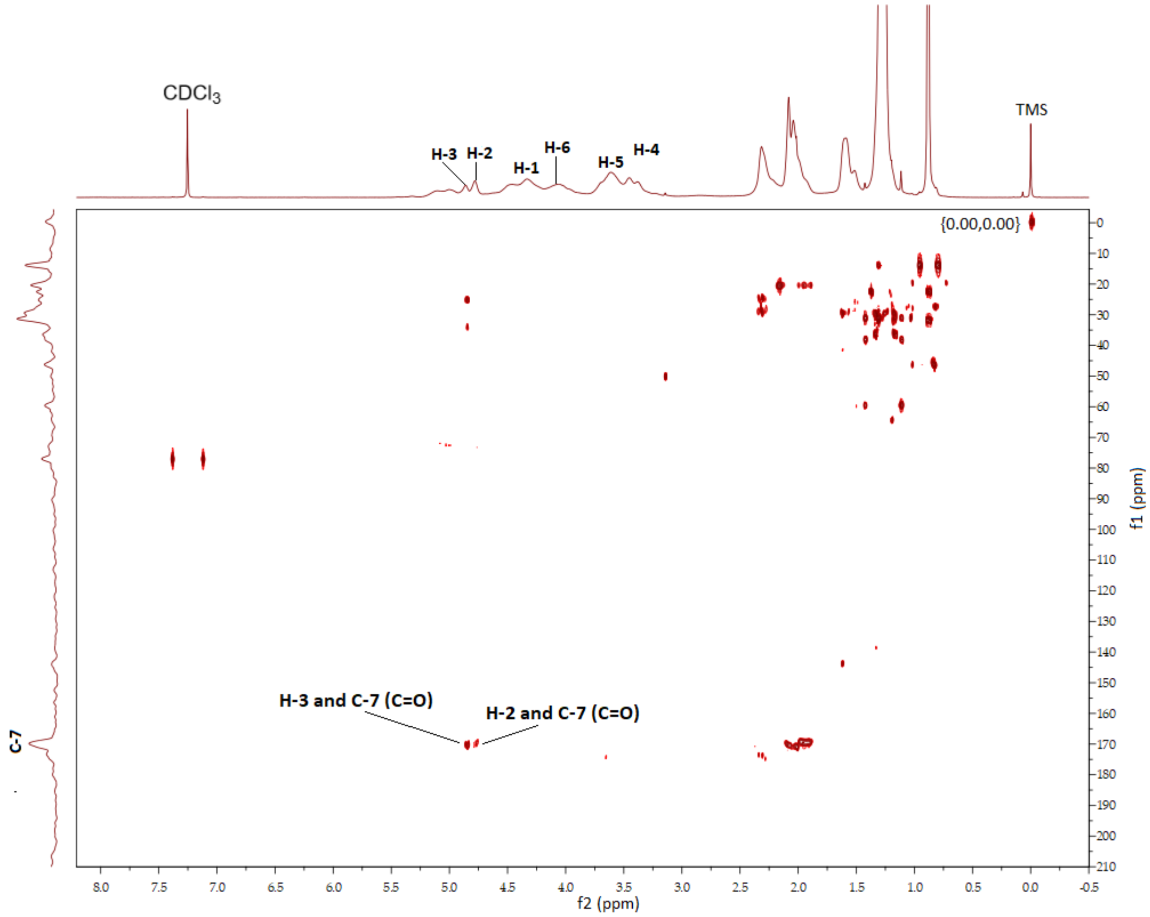


**Figure S8.** HMBC spectrum of Cellulose Palmitate (CP-3) in Chloroform-d (800 MHz Cryoprobe, 256 scans, 128 increments, 16 h acquisition time, 323 K).
